# Flaccidoxide-13-Acetate Extracted from the Soft Coral *Cladiella kashmani* Reduces Human Bladder Cancer Cell Migration and Invasion through Reducing Activation of the FAK/PI3K/AKT/mTOR Signaling Pathway

**DOI:** 10.3390/molecules23010058

**Published:** 2017-12-27

**Authors:** Choo-Aun Neoh, Wen-Tung Wu, Guo-Fong Dai, Jui-Hsin Su, Chih-I Liu, Tzu-Rong Su, Yu-Jen Wu

**Affiliations:** 1 Department of Research, Pingtung Christian Hospital, Pingtung 90059, Taiwan; neohca@gmail.com; 2 Department of Food Science and Nutrition, Meiho University, Pingtung 91202, Taiwan; gerrywu8769@yahoo.com.tw; 3 Department of Biological Technology, Meiho University, Pingtung 91202, Taiwan; fwind101@gmail.com; 4 National Museum of Marine Biology and Aquarium, Pingtung 94450, Taiwan; x2219@nmmba.gov.tw; 5 Department of Nursing, Meiho University, Pingtung 91202, Taiwan; x00003077@meiho.edu.tw; 6 Antai Medical Care Cooperation Antai Tian-Sheng Memorial Hospital, Pingtung 92842, Taiwan

**Keywords:** flaccidoxide-13-acetate, bladder cancer cells, migration, invasion, matrix metalloproteinase

## Abstract

Metastasis of cancer is the cause of the majority of cancer deaths. Active compound flaccidoxide-13-acetate, isolated from the soft coral *Cladiella kashmani*, has been found to exhibit anti-tumor activity. In this study, Boyden chamber analysis, Western blotting and gelatin zymography assays indicated that flaccidoxide-13-acetate exerted inhibitory effects on the migration and invasion of RT4 and T24 human bladder cancer cells. The results demonstrated that flaccidoxide-13-acetate, in a concentration-dependent manner, reduced the levels of matrix metalloproteinase-2 (MMP-2), MMP-9, urokinase-type plasminogen activator receptor (uPAR), focal adhesion kinase (FAK), phosphatidylinositide-3 kinases (PI3K), p-PI3K, AKT, p-AKT, mammalian target of rapamycin (mTOR), p-mTOR, Ras homolog gene family, member A (Rho A), Ras, mitogen-activated protein kinase kinase 7 (MKK7) and mitogen-activated protein kinase kinase kinase 3 (MEKK3), and increased the expressions of tissue inhibitor of metalloproteinase-1 (TIMP-1) and TIMP-2 in RT4 and T24 cells. This study revealed that flaccidoxide-13-acetate suppressed cell migration and invasion by reducing the expressions of MMP-2 and MMP-9, regulated by the FAK/PI3K/AKT/mTOR pathway. In conclusion, our study was the first to demonstrate that flaccidoxide-13-acetate could be a potent medical agent for use in controlling the migration and invasion of bladder cancer.

## 1. Introduction

Urothelial carcinoma, also called transitional cell carcinoma (TCC), is a malignant tumor arising from the transitional epithelium of the urinary tract [1]. TCC is the most common type of bladder cancer, and often occurs at the ureter or renal pelvis. Its mortality rate ranks second of all urinary tract cancers [2,3]. Studies of clinical cases have indicated that more than 90% of bladder cancers are TCC in origin [4,5], and the vast majority of deaths of patients with bladder cancer are due to invasive, metastatic TCC [6]. In the aging population, treatment of TCC is becoming more important, as an advanced age is known to increase the risk of bladder cancer [7]. Metastasis of TCC may arise from infiltration of the primary tumor to nearby tissue or through the systemic circulation to distal organs [8]. Treatment becomes more challenging when tumor metastasis occurs, and systemic therapy in the main, such as immune therapy or chemotherapy, is used to control the growth of metastatic cancer cells [9]. BCG is one of the most commonly-used intravesical perfusion treatments for the management of bladder cancer, as the BCG-induced host immune response may successfully destroy bladder cancer cells [10,11]. In recent years, a combination of chemotherapy and radiation for the treatment of invasive bladder cancer in patients who are not candidates for or decline cystectomy has been developed [12,13]. Metastasis is responsible for the majority of cancer deaths, and whether or not metastasis occurs or not is often seen as an index of treatment efficiency and disease severity in the care of cancer patients. Therefore, understanding the details of the molecular mechanism will assist in the development new drugs against cancer [14].

Marine soft corals are resources rich in bioactive materials, which possess bioactivities including anti-cancer, anti-fungus, anti-inflammation, anti-virus, ciguatoxin and cytotoxic properties [15]. Natural diterpenoids are common secondary metabolites of terrestrial and marine organisms, and cytotoxicity is one of the major features of compounds of this type [16,17,18,19]. In 2009, Hou and colleagues [20] found that ovatodiolide, a diterpenoid, induced reactive oxygen species (ROS) and down-regulated FLICE inhibitory protein (FLIP), which may stimulate apoptosis in oral squamous cells. In 2014, Huang and coworkers [21] reported that a diterpenoid compound, 5-episinuleptolide acetate (5EPA), isolated from the soft coral *Sinularia* sp., could induce apoptosis in leukemia cells through inhibition of Hsp90 protein [21]. The results of these studies suggested that diterpenoids have cytotoxicity against cancer cells.

**Figure 1:**
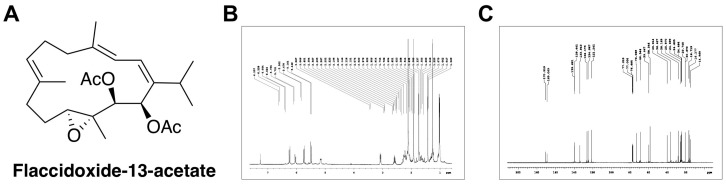
(**A**) Chemical structure of laccidoxide-13-acetate; (**B**) ^1^H NMR spectrum of flaccidoxide-13-acetate in CDCl_3_ at 400 MHz; (**C**) ^13^C NMR spectrum of flaccidoxide-13-acetate in CDCl_3_ at 100 MHz.

Flaccidoxide-13-acetate (Figure 1) is a cembrane-type diterpenoid derived from soft coral *Cladiella kashmani*, which has been shown to possess toxicity against a crustacean brine shrimp [22]. In this study, we utilized the natural bioactivity of flaccidoxide-13-acetate and investigated its effects on human bladder cancer cell migration and invasion. To the best of our knowledge, no study has previously applied flaccidoxide-13-acetate to human bladder cancer cells; the present study aimed to explore the mechanism and function of the anti-cancer activity, the results of which will form a useful reference for the development of new drugs for bladder cancer treatment.

## 2. Results

### 2.1. Inhibitory Effects of Flaccidoxide-13-Acetate on Cell Migration and Invasion

**Figure 2:**
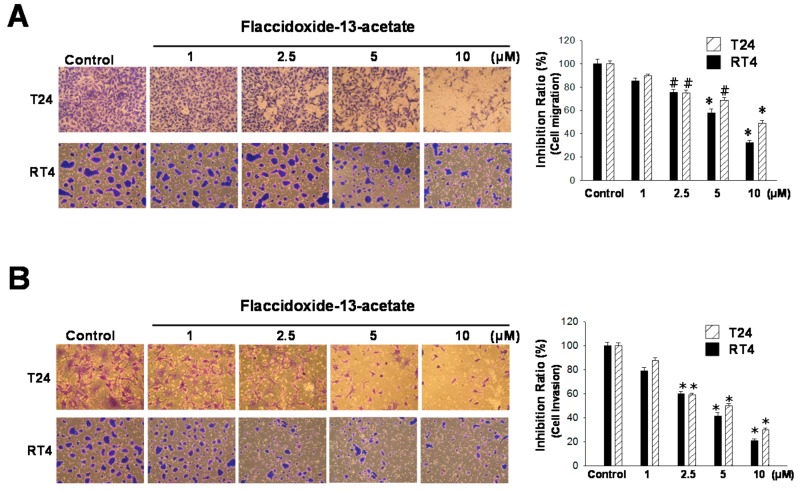
Flaccidoxide-13-acetate inhibits T24 and RT4 cell migration and invasion. (**A**) Effects of flaccidoxide-13-acetate on the inhibition of T24 and RT4 cell migration; (**B**) Effects of flaccidoxide-13-acetate on the inhibition of T24 and RT4 cell invasion. Control cells were treated with DMSO vehicle. Three independent experiments were performed for each assay. (^#^ *p* < 0.05, * *p* < 0.001).

Metastasis is a complicated tumor development process that includes cell migration and invasion. In this study, we employed a Boyden chamber to analyze the effects of flaccidoxide-13-acetate on the cell migration and invasion of T24 and RT4 bladder cancer cells. In terms of cell migration, when cells were incubated with increasing concentrations (1, 2.5, 5 and 10 μM) of flaccidoxide-13-acetate, cell migration was increasingly inhibited in both T24 and RT4 cells (Figure 2A). At 10 μM flaccidoxide-13-acetate, in comparison with cells treated with the control, only 48% and 32% of T24 and RT4 cells had migrated through the membrane, respectively. The results indicated that flaccidoxide-13-acetate inhibited bladder cancer cell migration. In terms of cell invasion, treatment with increasing concentrations of flaccidoxide-13-acetate also exerted an increasing inhibition effect on cell invasion in both T24 and RT4 cells (Figure 2B). At 10 μM flaccidoxide-13-acetate, T24 and RT4 cells that invaded through the membrane were only 38% and 25%, respectively, indicating that flaccidoxide-13-acetate reduced bladder cancer cell invasion.

### 2.2. Effects of Flaccidoxide-13-Acetate on the Expression Levels of MMP-2, MMP-9, uPAR, TIMP-1 and TIMP-2

**Figure 3:**
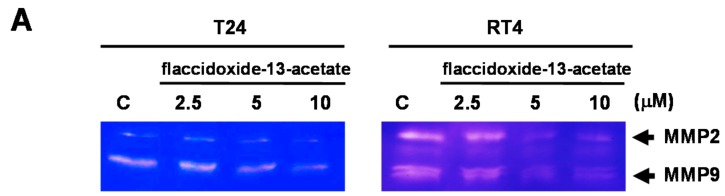

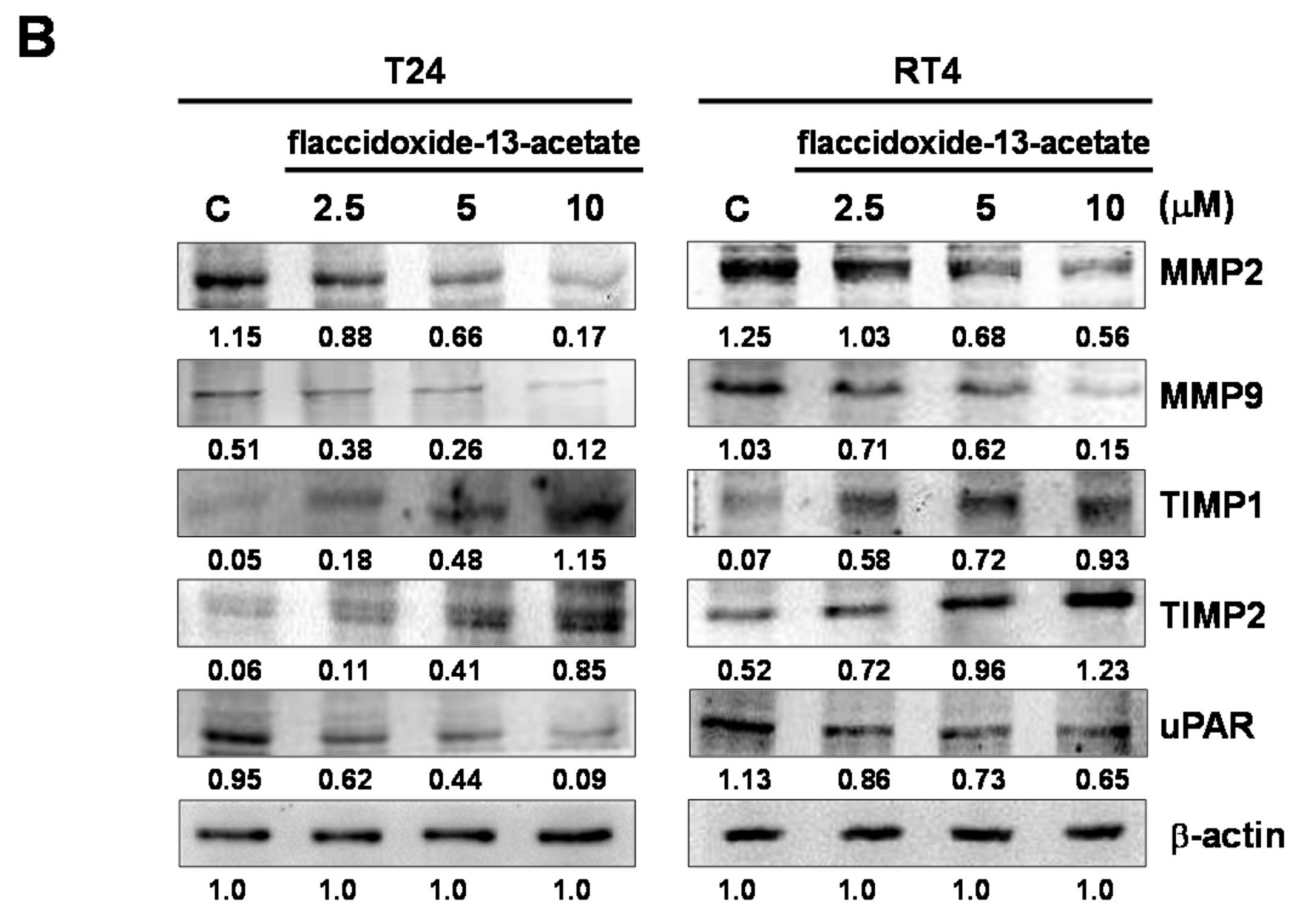
Flaccidoxide-13-acetate suppressed MMP-2, MMP-9 and uPAR protein expressions and augmented TIMP-1 and TIMP-2 protein expressions. (**A**) Gel zymography with gelatin was used to measure MMP-2 and MMP-9 activities (**B**) The expression levels of MMP-2, MMP-2/-9, uPAR and TIMP-1/-2 were analyzed by western blotting from total cell lysates of T24 and RT4 cells treated with flaccidoxide-13-acetate. Control (C) indicates cells treated with DMSO vehicle only. β-actin was used as the protein loading control.

MMP-2 and MMP-9 are members of the matrix metalloproteinase (MMP) protein family, and have the ability to cleave collagen and other types of extracellular matrix (ECM). Both MMP-2 and MMP-9 are known to be associated with metastasis and angiogenesis. The results of zymography showed that with increasing concentrations of flaccidoxide-13-acetate (2.5–10 μM), the activities of MMP-2 and MMP-9 decreased in both T24 and RT4 cells (Figure 3A). In addition, the results of western blotting also revealed that the protein expressions levels of MMP-2, MMP-9 and uPAR were increasingly inhibited upon treatment with increasing concentrations of flaccidoxide-13-acetate, while the levels of TIMP-1 and TIMP-2 were increased (Figure 3B).

### 2.3. Effects of Flaccidoxide-13-Acetate on the FAK/PI3K/AKT/mTOR Signaling Pathway

**Figure 4:**
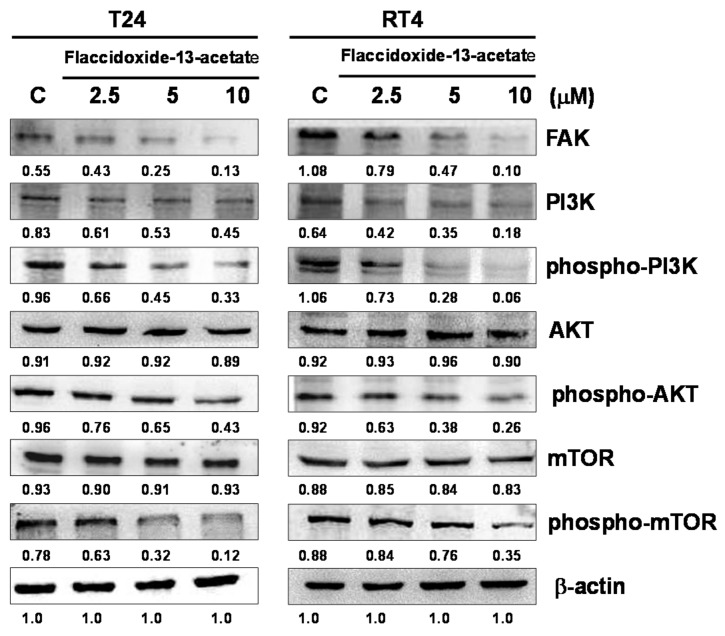
Flaccidoxide-13-acetate regulated the expression levels of mTOR signaling pathway proteins. Western blotting analysis demonstrated the changes in expressions levels of FAK, PI3K, phosphorylated-PI3K, AKT, phosphorylated-AKT, mTOR and phosphorylated-mTOR in T24 and RT4 cells treated with flaccidoxide-13-acetate. C: cells treated with DMSO vehicle only. β-actin was used as the protein loading control.

The FAK/PI3K/AKT/mTOR signaling pathway plays important roles in cell proliferation, differentiation, survival and tumor cell metastasis. In the present study, we used immunostaining to investigate the changes in FAK/PI3K/AKT/mTOR signaling protein expressions in T24 and RT4 cells after flaccidoxide-13-acetate treatment. The results showed that with an increasing flaccidoxide-13-acetate concentration, the levels of FAK, phosphorylated-PI3K, phosphorylated-AKT and phosphorylated-mTOR decreased in T24 and RT4 cells (Figure 4), suggesting that the inhibition effect of flaccidoxide-13-acetate on T24 and RT4 bladder cancer cells is associated with reduction of the activities of FAK/PI3K/AKT/mTOR signaling proteins.

### 2.4. Effects of Flaccidoxide-13-Acetate on the Expressions of Cell Migration- and Invasion-Associated Proteins

**Figure 5:**
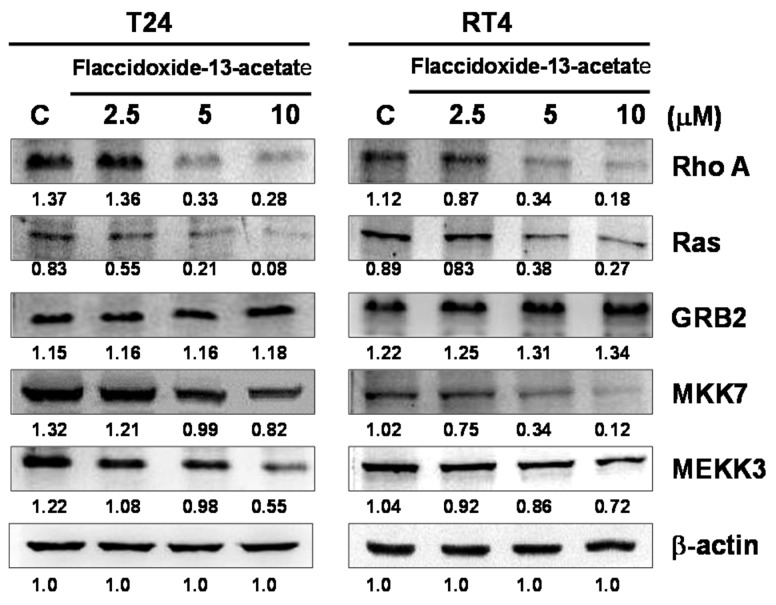
Flaccidoxide-13-acetate suppressed the expression levels of proteins associated with cell migration and invasion. Western blotting analysis was employed to examine the expression levels of RhoA, Ras, GRB2, MKK7 and MEKK3 in T24 and RT4 cells treated with flaccidoxide-13-acetate. C: cells treated with DMSO vehicle only. β-actin was used as the protein loading control.

To further understand the effects of flaccidoxide-13-acetate on the expressions of cell migration- and invasion-associated proteins such as RhoA, Ras, growth factor receptor-bound protein 2 (GRB2), mitogen-activated protein kinase kinase 7 (MKK7) and MAP kinase kinase kinase 3 (MEKK3), we utilized immunostaining to measure their expressions. As shown in Figure 4, with increasing concentrations of flaccidoxide-13-acetate, the expressions of Ras, RhoA, MEKK3 and MEKK7 were all suppressed (Figure 5).

### 2.5. Inhibition of PI3K Reduced the Cell Migration and MMP-2 and MMP-9 Protein Expression

**Figure 6:**
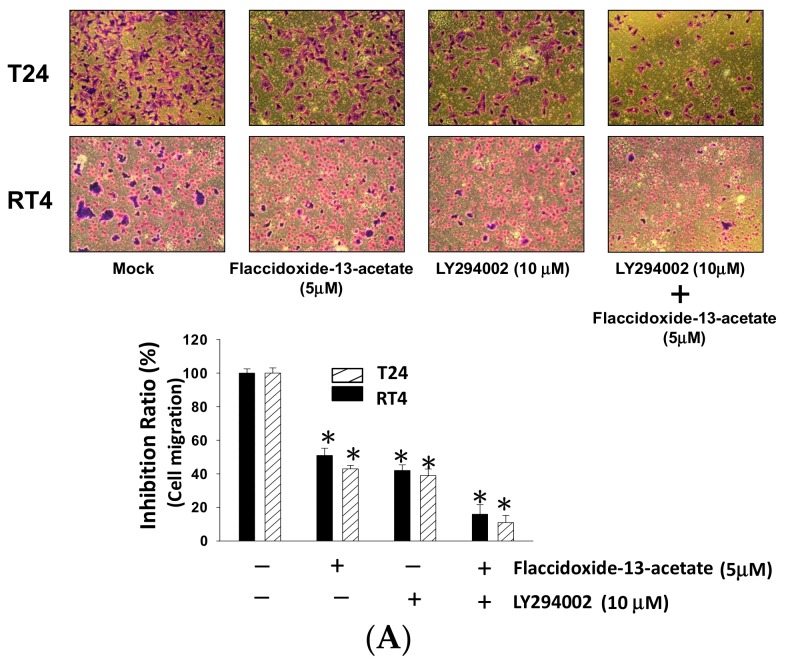

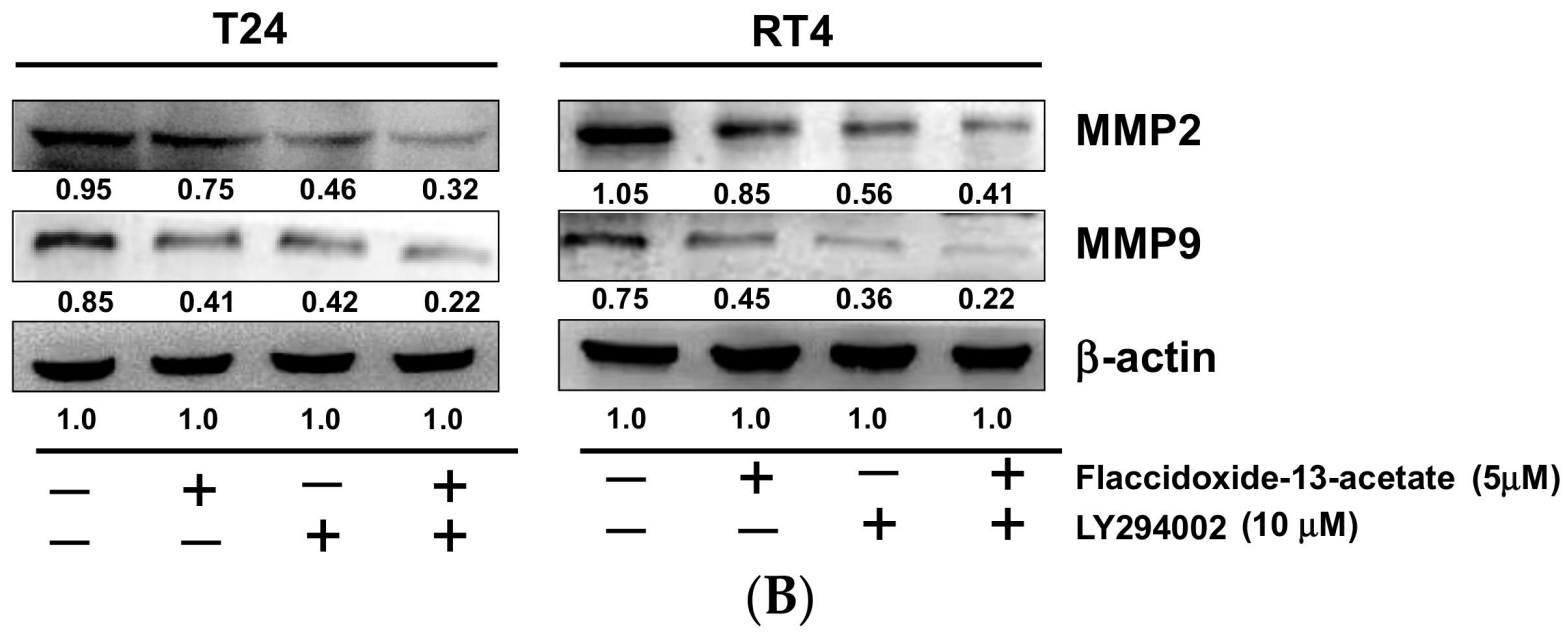
Inhibition of PI3K reduce the cell migration and MMP-2 and MMP-9 protein expression in T24 and RT4cells. (**A**) flaccidoxide-13-acetate and LY294002 inhibited T24 and RT4 cell migration and penetration through Transwell membranes. (* *p* < 0.001); (**B**) Western blotting showed the protein expression profiles of MMP-2 and MMP-9 in T24 and RT4 cells treated with flaccidoxide-13-acetate (5 μM) and LY294002 (10 μM). β-actin was used as the protein loading control.

To further examine the association between flaccidoxide-13-acetate with the aforementioned PI3K/AKT/mTOR pathways, LY292400, a PI3K inhibitor, was used to elucidate the effects on cell migration inhibited by flaccidoxide-13-acetate through PI3K/AKT/mTOR pathway. The results indicated that the cell migration of the flaccidoxide-13-acetate-treated T24 and RT4 cells reduced from 80% to 41% after treatment with LY292400 (10 mM) (Figure 6A). Furthermore, the expression levels of MMP-2 and MMP-9 were shown a significant reduction in flaccidoxide-13-acetate—treated T24 and RT4 cells with the addition of LY292400 (Figure 6B). Therefore, we proposed that the cell migration of T24 and RT4 cells should be suppressed by flaccidoxide-13-acetate through PI3K/AKT/mTOR pathway.

## 3. Discussion

Complications and other conditions caused by metastases are major causes of death from cancer. Direct causes may include tumors located in important organs, and indirect causes may include complications resulting from treatment to inhibit cancer cell growth and control metastasis. Therefore, in order to effectively control tumor metastasis, studies of the complex molecular mechanism of cancer metastasis are required [23]. We found that flaccidoxide-13-acetate at certain concentrations inhibited metastasis in T24 and RT4 bladder cancer cells, and investigated its possible mechanism of action.

Matrix metalloproteinases (MMPs) are a family of zinc-containing proteases that function to degrade extracellular matrix proteins, such as collagen, laminin and proteoglycan [24]. Using MMP-9-deficient mice, Itoh and coworkers [25] confirmed that MMP-9 mediates the process of tumor metastasis. In addition, Zheng et al. [26] found that the expressions of MMP-2, MMP-9 and VEGF were increased with increasing tumor size and depth of invasion. Tissue inhibitors of metalloproteinases (TIMPs) are inhibitors of MMPs. Therefore, study of the changes of MMP and TIMP proteins may help to further our understanding of the effects of drug treatment on the metastasis of cancer cells [27]. Furthermore, uPAR, the receptor of urokinase-type plasminogen activator (u-PA), has been shown to be highly-expressed in many tumor cells, and can be used as a predictor of cancer development [28]. In the present study, the results of cell migration and invasion experiments also demonstrated that the expressions and enzyme activities of MMP-2 and MMP-9 decreased with an increasing flaccidoxide-13-acetate concentration in both T24 and RT4 cells, while the expression levels of TIMP-1 and TIMP-2 increased with an increasing flaccidoxide-13-acetate concentration. In addition, the expression of uPAR was also regulated by flaccidoxide-13-acetate treatment (Figure 2). The aforementioned results suggested that flaccidoxide-13-acetate has the potential to inhibit bladder cancer metastasis.

MMP-2 and MMP-9 of the MMP family have been thought to play important roles in the processes of tumor invasion and metastasis [29]. Several studies have shown that FAK/PI3K/AKT/mTOR signaling is involved in cell proliferation, differentiation, survival and tumor metastasis, and the signaling pathway is also involved in the regulation of MMP-2 and MMP-9 [30,31,32]. We used immunostaining to examine whether flaccidoxide-13-acetate affects bladder cancer cell invasion and metastasis via the FAK/PI3K/AKT/mTOR signaling pathway. The expressions of FAK, non-phosphorylated and phosphorylated PI3K and non-phosphorylated and phosphorylated mTOR all decreased in T24 and RT4 bladder cancer cells with an increasing flaccidoxide-13-acetate concentration (Figure 3). Our results demonstrated that the specific PI3K inhibitor, LY294002, significantly suppressed the cell migration, and markedly inhibited the MMP-2 and MMP-9 proteins expression (Figure 6). Based on the results, we speculated that flaccidoxide-13-acetate may affect the ability of cancer cells to metastasize by inhibiting the FAK/PI3K/AKT/mTOR signaling pathway. Similarly, the results of a recent study by Wu et al. [33] showed that the active compound sinulariolide, isolated from cultured soft coral *Sinularia flexibilis*, inhibited the migration and invasion of hepatocellular carcinoma cells, also through the FAK/PI3K/AKT/mTOR pathway, by inhibiting the expressions of MMP-2 and MMP-9.

In the current study, we found that flaccidoxide-13-acetate inhibited the invasion and metastasis of T24 and RT4 bladder cancer cells, and the inhibition effect proceeded in a concentration-dependent manner. Furthermore, tumor invasion- and metastasis-associated proteins, such as RhoA, Ras, MKK7 and MEKK3, were found to be inhibited by flaccidoxide-13-acetate treatment. A study by Lin et al. [34] found that curcumin treatment reduced the expressions of RhoA, MMP-2 and MMP-9 in mouse–rat hybrid retina ganglion cells (N18). In fact, previous study of human vascular endothelial cells (HMEC-1) demonstrated that RhoA can induce MMP-9 expression and promote endothelial cell invasion [35].

Based on the findings of our study and those of others, we inferred that RhoA acts as an upstream regulatory protein of MMPs, and when the expression of RhoA is inhibited by flaccidoxide-13-acetate, the expressions of MMP-2 and MMP-9 decrease, leading to reductions in the cell migration and invasion abilities of T24 and RT4 bladder cancer cells (Figure 4). In addition, the expression of Ras is also inhibited by flaccidoxide-13-acetate. The results of a study by Thant et al. [36] also confirmed that the tumor invasion ability of cells is mediated by MMP-2 expression, which is regulated by Ras signaling, and thus inhibition of Ras affects the activation of MMP-2, and subsequently alters the invasiveness of tumor cells.

## 4. Material and Methods

### 4.1. Chemicals and Reagents

Streptomycin, penicillin and Dulbecco’s modified Eagle’s medium, fetal bovine serum (FBS) were purchased obtained from Gibco BRL (Grand Island, NY, USA). Flaccidoxide-13-acetate was extracted from the soft coral *Cladiella kashmani*, following a protocol published previously [22]. The compound was dissolved in dimethyl sulfoxide (DMSO). 3-(4,5-dimethylthiazol-2-yl)-2,5-diphenyltetrazolium bromide (MTT), rabbit anti-human β-actin antibody and other general chemicals were obtained from Sigma-Aldrich Corporation (St. Louis, MO, USA). Chemiluminescent substrate for western HRP development was obtained from Pierce (Rockford, IL, USA). Goat anti-rabbit IgG conjugated horseradish peroxidase, rabbit antibodies against human FAK, mTOR, phosphorylated-mTOR, MEKK3, MKK7, RhoA and GRB2 were purchased from Epitomics Inc. (Burlingame, CA, USA). Rabbit antibodies against human TIMP-1 and TIMP-2 were purchased from ProteinTech Group Inc. (Rosemont, IL, USA). Rabbit antibodies against human PI3K, phosphorylated-PI3K, uPAR, MMP-2and MMP-9 were purchased from Cell Signaling Technology Inc. (Danvers, MA, USA).

### 4.2. Cell Culture

Human bladder cancer T24 and RT4 cells were purchased from the Taiwan Food Industry Research and Development Institute (Hsinchu, Taiwan) and grown in Dulbecco’s modified Eagle’s medium (containing 10% (*v*/*v*) FBS, 100 μg/mL streptomycin and 100 units/mL penicillin,) at 37 °C in a 5% CO_2_ incubator. When examining the effects of flaccidoxide-13-acetate, cells were treated with vehicle (DMSO) as a control. Cells were treated with various concentrations of flaccidoxide-13-acetate and incubated for 24 h before harvesting for further analyses.

### 4.3. MTT Assay

Cell viability was assessed by the MTT assay [37]. To determine whether flaccidoxide-13-acetate treatment was cytotoxic, cells placed in 24-well plates were treated with flaccidoxide-13-acetate (at 1, 2.5, 5 and 10 μM). After 24 h of incubation, 50 μL of MTT (1 mg/mL in PBS) were added, followed by further incubation for 3 h; then, the culture medium was removed, and the cells were dissolved in DMSO and shaken for 10 min. The plates were then analyzed using a microplate reader (Bio-Rad; Hercules, CA, USA). All experiments were repeated three times.

### 4.4. Cell Migration and Invasion Assays

The cell migration assay was performed using a method described previously by Neoh et al. [38]. Briefly, 5 × 10^4^ cells placed into a Boyden chamber (Neuro Probe; Cabin John, MD, USA) in serum-free media were treated with or without flaccidoxide-13-acetate, then incubated for 24 h at 37 °C to allow migratory cells to pass through the membrane. The cell invasion assay was performed using a protocol described previously by Yeh et al. [39]. Briefly, each well of a Transwell insert with 8 μm-pore-size polycarbonate membrane filters was coated with 0.5 mg/mL of Matrigel, following which the cell suspension was placed into the upper chamber with serum-free media, and cell culture medium supplemented with serum was added into the bottom chamber. At the end of incubation, the membranes through which cells had migrated and invaded into the lower chamber were fixed with methanol and stained with 5% Gimmsa (Merck; Darmstadt, Germany), then counted under a light microscope.

### 4.5. Determination of MMP-2/-9 Activities by Gelatin Zymography

The activities of MMP-2/-9 in conditioned media were measured using gelatin zymography, as described previously by Chen et al. [40]. Briefly, cells were incubated for 24 h with different concentrations of flaccidoxide-13-acetate, and the culture media were collected. Samples were separated by 10% SDS-PAGE containing 0.2% gelatin under non-reducing conditions. Separation was achieved, and the gels were washed three times in washing buffer (100 mM NaCl, 2.5% Triton-X 100, 50 mM Tris-HCl, pH = 7.5), then activated in reaction buffer (200 mM NaCl, 1 mM CaCl_2_, 0.02% NaN_3_, 1 µM ZnCl_2_, 2% Triton-X 100, 50 mM Tris-HCl buffer, pH = 7.5) for 24 h at 37 °C. Finally, the gels were stained (Coomassie Blue R-250) then de-stained.

### 4.6. Immunoblotting Analysis

After stimulation, cells from each well were rinsed twice with ice-cold PBS and lysed in 100 μL of cell lysis buffer (20 mM Tris/HCl pH 7.5, 125 mM NaCl, 1% Triton X-100, 1 mM MgCl_2_, 25 mM β-glycerophosphate, 50 mM NaF, 100 μM Na_3_VO_4_, 1 mM PMSF, 10 μg/mL leupeptin, 10 μg/mL aprotinin). Protein lysates were denatured in SDS, electrophoresed on 10% SDS-PAGE, and transferred onto PVDF membrane. Non-specific binding was blocked with TBST (50 mM Tris-HCl pH 7.5, 150 mM NaCl, 0.1% Tween 20) containing 5% nonfat milk for 1 h at room temperature. After incubation with the appropriate first antibody, the membrane was washed three times with TBST, followed by incubation with a secondary antibody for 1 h. After three washes with TBST, protein bands were detected using ECL reagent. The western blot data were quantified with Image J software (NIH, Bethesda, MD, USA).

### 4.7. Statistical Evaluation

Data were collected from at least three experiments, which were performed in duplicate, and values are expressed as the mean ± S.E.M. Analysis of variance (ANOVA) was used to assess the statistical significances of differences, and *p* < 0.05 was considered to indicate statistical significance.

## 5. Conclusions

**Figure 7:**
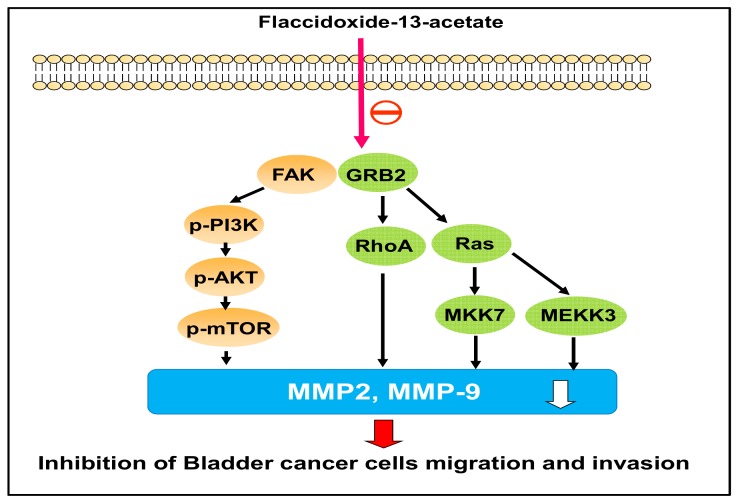
Proposed signaling pathways for flaccidoxide-13-acetate is mediated inhibition of bladder cancer cell migration and invasion. The effect of flaccidoxide-13-acetate is down-regulation MMP-2 and MMP-9 expression through FAK/PI3K/AKT/mTOR pathway signaling pathways.

In conclusion, this study demonstrated that flaccidoxide-13-acetate inhibits the migration and invasion of T24 and RT4 bladder cancer cells through the down-regulation of MMP-2 and MMP-9 expressions, and the process is controlled by the FAK/PI3K/AKT/mTOR pathway (Figure 7). Our findings suggested that flaccidoxide-13-acetate has the potential to be developed as new drug for control of the invasion and metastasis of bladder cancer cells.
